# A new method to determine tissue specific tissue factor thrombomodulin activities: endotoxin and particulate air pollution induced disbalance

**DOI:** 10.1186/1477-9560-6-14

**Published:** 2008-10-01

**Authors:** Kim Frederix, Ingeborg M Kooter, René van Oerle, Diane Fens, Karly Hamulyak, Miriam E Gerlofs-Nijland, Hugo ten Cate, Henri MH Spronk

**Affiliations:** 1 Department of Internal Medicine, Laboratory for Clinical Thrombosis and Haemostasis, Cardiovascular Research Institute Maastricht, Maastricht University, Maastricht, The Netherlands; 2 Department of Internal Medicine, Division of Haematology, University Hospital Maastricht, Maastricht, The Netherlands; 3 Centre for Environmental Health Research, National Institute for Public Health and the Environment, Bilthoven, The Netherlands

## Abstract

**Background:**

Increase in tissue factor (TF) and loss in thrombomodulin (TM) antigen levels has been described in various inflammatory disorders. The functional consequences of such changes in antigen concentrations in the coagulation balance are, however, not known. This study was designed to assess the consequences of inflammation-driven organ specific functional properties of the procoagulant response.

**Methods:**

Tissue specific procoagulant activity was assessed by adding tissue homogenate to normal human pool plasma and recording of the thrombin generation curve. The new technique was subsequently applied on two inflammation driven animal models: 1) mouse lipopolysaccharide (LPS) induced endotoxemia and 2) spontaneously hypertensive rats exposed to environmental air pollution (particulate matter (PM).

**Results:**

Addition of lung tissue from untreated animals to human plasma suppressed the endogenous thrombin potential (ETP) (175 ± 61 vs. 1437 ± 112 nM.min for control). This inhibitory effect was due to TM, because a) it was absent in protein C deficient plasma and b) lungs from TM^pro/pro ^mice allowed full thrombin generation (ETP: 1686 ± 209 nM.min). The inhibitory effect of TM was lost after LPS administration to mice, which induced TF activity in lungs of C57Bl/6 mice as well as increased the ETP (941 ± 523 vs. 194 ± 159 nM.min for control). Another pro-inflammatory stimulus, PM dose-dependently increased TF in the lungs of spontaneously hypertensive rats at 4 and 48 hours after PM exposure. The ETP increased up to 48 hours at the highest concentration of PM (1441 ± 289 nM.min vs. saline: 164 ± 64 nM.min, p < 0.0001), suggesting a concentration- and time dependent reduction in TM activity.

**Conclusion:**

Inflammation associated procoagulant effects in tissues are dependent on variations in activity of the TF-TM balance. The application of these novel organ specific functional assays is a useful tool to monitor inflammation-driven shifts in the coagulation balance within animal or human tissues.

## Introduction

The inflammation associated procoagulant response is a characteristic feature of innate immunity and a reflection of the crosstalk between inflammation and blood coagulation. During acute inflammatory conditions, such as sepsis, the procoagulant response is characterized by increased cellular expression of tissue factor (TF) [1-3], the physiological trigger of coagulation. In addition, a loss of endogenous anticoagulant activity is proposed to occur during inflammation, mainly based on *in vitro *studies showing loss in thrombomodulin (TM) and endothelial protein C receptor (EPCR) antigen in cultured endothelial cells [4-6]. In addition, incidental clinical studies have documented reduced levels of circulating TM antigen during bacterial sepsis [7].

In chronic inflammatory diseases, including atherosclerosis and cancer, increased expression of TF and reduced expression of TM have also been observed [8-11]. In such chronic conditions, a disturbed balance in pro- and anticoagulant activities is thought to have an unfavourable effect on disease activity [11]. In atherosclerosis, increased expression of TF and reduced anticoagulant activity may stimulate thrombogenicity. In cancer, the loss of TM has been linked to loss of cell differentiation, resulting in increased tumour cell activity [12,13].

Recently, inflammation induced by environmental particulate air pollution (particulate matter: PM) has been linked to chronic morbidity including cardiovascular and lung disease, as well as mortality in several large clinical studies [14-18]. The perturbation of the balance between pro- and anticoagulant activity in organs exposed to PM, however, may be a contributing factor to development of disease.

Despite the knowledge about inflammation induced changes in antigen levels of TM and TF, the overall functional changes in pro- and anticoagulant activities have not been studied. Since multiple factors are thought to be involved in disorders as atherosclerosis, cancer, and PM induced chronic lung disease, an overall analysis of changes in the balance between pro- and anticoagulant proteins would be helpful in assessing the net effects of compounds that modulate inflammation and/or coagulation. We therefore hypothesized that exposure to PM stimulates blood coagulation by an indirect inflammation mediated pathway, thereby disturbing the tissue factor/thrombomodulin balance in favour of a procoagulant phenotype.

To assess the procoagulant phenotype of tissues, e.g. the balance between TF and TM activity, we first developed a new functional assay in which the activity of both proteins is indirectly determined through in vitro thrombin generation. Second, the method was validated using a mouse endotoxemia model. Finally, the new method was applied to study the PM induced procoagulant phenotype in a rat model.

## Materials and methods

### Study design

Three different studies were undertaken to study the effects of inflammation on tissue-specific hypercoagulability: 1. *Development of a new method to assess TF and TM activity in tissue homogenates*. In the first study a new method was developed to determine the activities of TF and TM within tissue homogenates. To this purpose, a thrombin generation assay was modified to allow the addition of tissue homogenates. 2. *Validation of the new method using a mouse endotoxemia model*. The new developed method to determine the activities of TF and TM within tissue homogenates was validated using a mouse LPS-induced endotoxemia model. 3. *Effects of particulate matter on tissue factor activity and thrombin generation*. The inflammation-driven procoagulant effects of particulate matter exposure on lung tissue were studied using a spontaneously hypertensive rat model.

### Mouse strains and LPS-induced endotoxemia

C57Bl/J6 mice (Charles River, Someren, The Netherlands) were used as wild type (WT) control mice as well as in the LPS induced endotoxemia model. TM^pro/pro ^mice carry a single amino acid substitution (Glu404>Pro) in the TM protein, which causes a reduced expression of TM and disrupts the TM-dependent activation of protein C for almost 90% [19].

Endotoxemia was induced by intraperitoneal (i.p.) injection of 2 mg LPS (E. coli serotype O55:B5; Sigma-Aldrich, St. Louis, MO) per kg bodyweight. After 6 hours, mice were anaesthetized with 4% isoflurane and blood was drawn from the vena cava after injection of 7 % of the bodyweight 3.2% citrate buffer as described previously [20]. Mice were perfused using St. Thomas cardioplegic solution (University Hospital Maastricht, The Netherlands) through the portal vein for 10 minutes. Lung, liver, heart, kidney, brain, and spleen were snap frozen in liquid nitrogen. All mice were 10–12 weeks old and n = 6 mice per group were used. All mice studies were approved by the Animal Care and Use Committee of the Maastricht University.

### Rat experiments and particulate matter

Eleven to twelve weeks old spontaneously hypertensive (SHR) male rats were exposed to PM by intratracheal instillation [21]. In brief, rats were anaesthetized with 4% halothane and road tunnel dust (RTD, solved in saline at concentrations of 0.15, 0.5, 1.5 or 5 mg/ml) was instilled in a volume of 2 ml/kg body weight to the final concentrations of 0.3, 1, 3, and 10 mg/kg body weight [21]. In addition, rats were instilled with either saline solution (0.9% NaCl), or urban dust preparation EHC-93 (urban dust PM sample collected from Environmental Health Centre in 1993, Ottawa, Canada) at a final concentration of 10 mg per kg body weight through a cannula inserted into the trachea just above the bifurcation. Road tunnel dust (RTD) and urban air PM sample (Ottawa dust; EHC-93) were chemically characterized and described previously [21]. Briefly, RTD is an integrated PM sample, consisting of coarse and fine fractions, collected at the exit of a motorway tunnel at Hendrik-Ido-Ambacht (HIA). This was collected on polyurethane foam (PUF) using a high volume cascade impactor (HVCI) [22]. EHC-93, an urban air PM sample recovered by vacuuming of bag-house filters of the Environmental Health Centre in Ottawa in Canada was used as a second ambient PM sample. The chemical composition and biological reactivity of EHC-93 have been described earlier [23,24].

Experiments were approved by the Animal Ethics Committee (IUCAC) of the Dutch National Vaccine Institute (NVI), Bilthoven, The Netherlands.

### Tissue preparation

Tissues were freeze dried for three days. The dried tissues were pulverized using a small mortar and divided into two portions for either mRNA isolation or protein analysis. Fractions for protein analyses were dissolved in 50 mM n-octyl β-D-glucopyranoside (Sigma-Aldrich) in HN-Buffer (25 mM HEPES, 175 mM NaCl, pH 7.7), vortexed, and centrifuged twice (10 min, 13000 rpm). Total protein content of the tissue homogenates was spectrophotometrically determined using the Biorad DC Protein Assay system according to the manufacturer's instructions (Bio-Rad Laboratories B.V., Veenendaal, The Netherlands).

### Thrombin-antithrombin (TAT) levels

Plasma levels of thrombin-antithrombin complexes (TAT) were measured by a specific murine sandwich TAT ELISA as described previously [20].

### mRNA quantification

Total RNA was isolated using a Trizol method according to the single-step method previously described [25], with minor modifications (Sigma-Aldrich). Five to ten mg freeze-dried tissue was dissolved in 1 mL Tri Reagent. Samples were stored at -80° for precipitation steps, RNA was washed with 80 %(v/v) ethanol and concentrations were spectrophotometrically measured in RNase free water. cDNA was synthesised using the Avian Enhanced First Strand Synthesis kit (Sigma-Aldrich) according to the manufacturer's instructions. mRNA levels for TF and TM were measured on a Light cycler system 1.2 (Roche, Woerden, The Netherlands) using the SYBR premix Ex Taq kit (Takara Bio Inc., Shiga, Japan) according to the manufacturer's instructions and using the following primers: TM forward 5'-GTCACGGTCTCGACAG, TM reverse 5'-GCAGCGTTTGAAAGTCC, TF forward 5'-GAAGAACACCCCGTCG, and TF reverse 5'-GTTCGTCCTAACGTGACA. Quantification was done relative to the household gene glyceraldehyde 3 phosphate dehydrogenase (GAPDH) using forward 5'-TCCCAGAGCTGAACGG and reverse 5'-GAAGTCGCAGGAGACA primers.

Levels of TF and TM are expressed as ratios of TF/GAPDH or TM/GAPDH and each data point represents an average of 3 measurements.

### Tissue factor activity measurement

TF activities in tissue homogenates were determined using a home-made activity assay as previously described [26]. In brief, dissolved tissue homogenates with a concentration of 1 mg/mL total protein were diluted 20 times (brain, lung and aorta) or 5 times (all other tissues) in HN-buffer. A reference curve was prepared from Innovin (Dade Behring Holding GmbH, Liederbach, Germany), starting with 5 pM and diluted serially 7 times, also in HN buffer. Samples were incubated for 45 minutes at 37°C in the presence of recombinant factor VII (FVII) (Novo Nordisk, Bagsværd, Denmark), 0.2 mM 20/80 PS/PC vesicles, 1 U/mL Bovine factor X (Sigma-Aldrich) and 100 mM Ca^2+^. The formation of factor Xa was then measured kinetically using the chromogenic substrate S 2765 (Chromogenix, final concentration of 0.7 mg/mL diluted in 50 mM Tris-HCl, 175 nM NaCl, 30 mM Na_2_EDTA, pH 7.4) by measuring the OD at 405 nm each 15 seconds, for 15 minutes at 37°C.

### Calibrated Automated Thrombogram

The Calibrated Automated Thrombogram (CAT, Thrombinoscope, the Netherlands) was used to determine the contribution of mouse and rat tissue homogenates to thrombin generation. We adapted the protocol from the recording of thrombin generation curves in platelet poor plasma as described previously [27]: 15 μl of tissue homogenate (5 mg/mL total protein) was added to 80 μL of platelet poor pooled human plasma (normal pool plasma, NPP) (University Hospital Maastricht), which consisted of plasma from 80 healthy volunteers. Thrombin generation was either triggered by adding 5 pM TF, 4 μM phospholipids (PL) (TF/PL) and 16.7 mM Ca^+2 ^to the reaction mixture, or without addition of TF/PL. In the absence of additional TF/PL triggering of thrombin generation depends entirely on procoagulant molecules, such as TF, present in the tissue homogenate. Furthermore, activation of the coagulation cascade via the contact system will occur in case no additional TF/PL are added and TF is not present in the tissue homogenate added. The following five parameters were derived from a thrombin generation curve: lag time, time to peak, peak height, and endogenous thrombin potential (ETP, the area under the curve). The lag time is merely determined by TF and factor VII, whereas both peak height and ETP are dependent on fibrinogen, prothrombin, and antithrombin (Dielis, Spronk et al. unpublished data). Both mouse and human active site inhibited FVIIa were a kind gift of Dr. Petersen (Novo Nordisk A/S, Denmark).

### Statistical analysis

Data are presented as mean with standard deviation. Differences between groups were assessed using non-paired Student's *t-*test for normal distribution or Mann-Whitney U test when distribution was not normal. P values < 0.05 were considered statistically significant. Statistical analyses were performed using SPSS version 12.01 for Microsoft Windows.

## Results

### Development of a new method to assess TF and TM activity in tissue homogenates

To assess the overall procoagulant activity of various organs, tissue homogenates were analyzed using an *in vitro *plasma-based fluorogenic thrombin generation assay. This method uses TF (5 pM), phospholipids (PL, 4 μM) (the so called PPP-reagent), and CaCl_2 _(16.7 mM) to trigger thrombin generation in platelet-poor human plasma. From the resulting thrombin generation curve several parameters can be derived, including the lag time (defined as the time to reach 1/6 of the maximum thrombin formed), the peak height (the maximum amount of thrombin formed), and the area under the curve, also known as the endogenous thrombin potential (ETP). For normal human pool plasma a lag time of thrombin generation of 2.56 ± 0.09 min and an ETP of 1592 ± 67 nM.min were observed.

**Figure 1:**
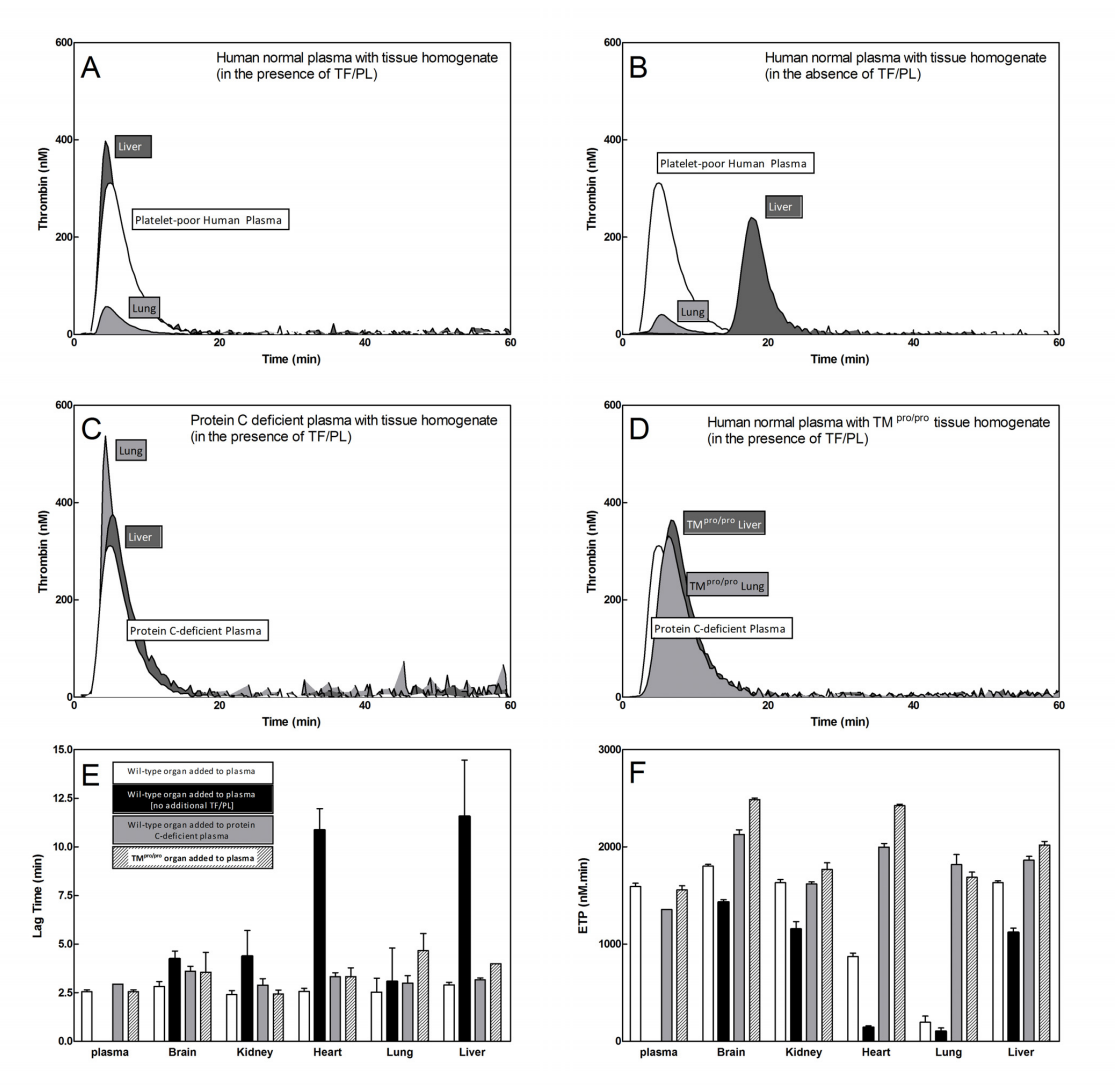
**Influence of lung and liver tissue homogenate on thrombin generation in plasma.** Lung (light grey curve) or liver (dark grey curve) homogenates were added to human normal plasma with additional TF (5 pM) and PL (4 μM) (Panel A) or in the absence of additional TF and PL (Panel B). For comparison, thrombin generation in human normal plasma triggered with 5 pM TF and 4 μM PL is plotted (white curve) in panels A, B, C, and DPanel C: Addition of lung (light grey curve) or liver (dark grey curve) homogenate to protein C deficient plasma (Panel C) in the presence of additional TF/PL. Panel D: Addition of lung (light grey curve) or liver (dark grey curve) homogenates from TM^pro/pro ^transgenic animals to platelet-poor human plasma. Panels E and F: overview of the lag time and ETP derived from thrombin generation curves for tissue homogenates added to platelet-poor human plasma in de absence of additional TF/PL (white bars) or in the presence of additional TF/PL (black bars), tissue homogenates added to protein C-deficient plasma (grey bars), and tissue homogenates from TMpro/pro transgenic animals to platelet-poor human plasma (hatched bars). Bars represent mean ± SD from 6 tissue homogenates.

Modification of this method consisted of the addition of normal mice tissue homogenate to platelet-poor human plasma, along with the additional reagent (5 pM TF, 4 μM, 16.7 nM CaCl_2_) to trigger thrombin generation. By doing so, addition of lung homogenate (ETP: 194 ± 159 nM.min) caused a 8-fold reduction in thrombin generation compared to platelet-poor human plasma alone (ETP: 1591 ± 67 nM.min, p < 0.01, Figure 1, Panel A). Furthermore, compared to platelet-poor human plasma alone heart homogenate (ETP: 874 ± 75 nM.min, p < 0.01) attenuated thrombin generation, whereas the addition of brain (ETP: 1802 ± 43 nM.min), kidney (ETP: 1631 ± 81 nM.min), liver (ETP: 1631 ± 49 nM.min), or spleen (ETP: 1298 ± 202 nM.min) had no significant influence (Figure 1F, white bars).

**Figure 2:**
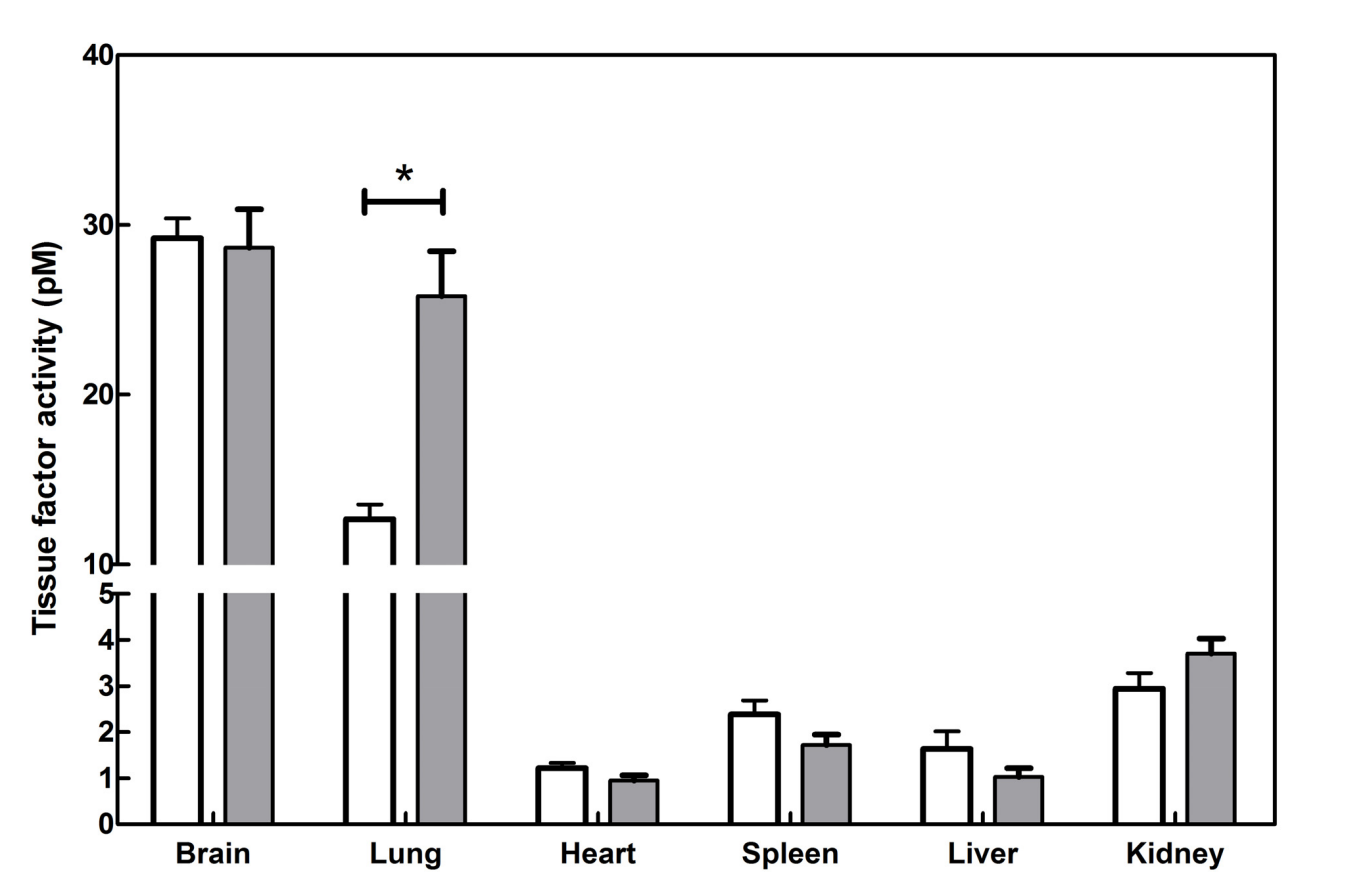
**TF activity in tissue homogenates before (white bars) and 6 hours after LPS administration (grey bars). **Tissue factor activity in brain, lung, heart, spleen, liver and kidney homogenates were measured at a total homogenate protein concentration of 1 mg/ml. Bars indicate mean ± SD of n = 6 animals per groups. * denotes p < 0.05.

As stated before, thrombin generation dependents on the addition of TF, PL, and Ca^2+^, although in the presence of only PL and Ca^2+^, thrombin generation can occur as a result of contact activation, which is characterized by a long lag time before thrombin generation (>10 minutes). If, however, tissue homogenates were added without additional TF, PL, and Ca^2+^, thrombin generation in platelet-poor human plasma became dependent on the presence of TF and PL within the added tissue homogenate. Upon addition of mouse tissue homogenates to plasma, thrombin generation in the absence of additional TF, PL, and Ca^2+^, showed considerable variation in lag times and ETPs compared to analysis in the presence of TF, PL, and Ca^2+ ^(Figure 1B, E, and 1F). The short lag times observed for the thrombin generation curves after addition of lung, brain, and kidney were comparable between analysis in the presence or absence of additional TF, PL, and Ca^2+ ^(Figure 1E), suggesting that sufficient amounts of TF were present in these tissues to trigger thrombin generation. These findings are compatible with the notion that the constitutive concentrations of TF are high in brain and lung, which is in agreement with the observed TF activity in the chromogenic assay (Figure 2). In contrast, liver (Figure 1B) and heart produced markedly prolonged lag times in the absence of additional TF, PL, and Ca^2+^, as compared to lung, brain, and kidney (Figure 1E), indicating low TF-concentrations in these homogenates and suggesting that contact activation triggered thrombin generation. This was supported by the observation that no thrombin generation was observed in the presence of the coagulation factor XIIa inhibitor corn trypsin inhibitor (CTI), or when FXII-deficient plasma was used instead of normal plasma (data not shown). The strongest overall procoagulant effect, expressed as ETP, was observed for liver, brain, and kidney, while lung and heart hardly induced any thrombin generation (Figure 1D). Kidney, liver, and spleen homogenates also initiated thrombin generation, as indicated by the ETP-values (Figure 1D), despite the rather low levels of endogenous TF (Figure 2).

To ensure that murine TF activity was indeed an important determinant of thrombin generation in platelet-poor human plasma, a separate experiment was performed in which active site inhibited factor VIIa (ASIS) was added to plasma containing mouse lung homogenate. In all cases, both human and murine ASIS prolonged the lag time dose dependently, impairing thrombin generation completely at the highest dose of ASIS (data not shown), demonstrating that TF from mouse tissue homogenates triggers thrombin generation in platelet-poor human plasma and that species specificity is not a limitation in this new method.

Overall, despite the presence of relatively large amounts of endogenous TF (Figure 2: TF activity in lung homogenate and Figure 1B lag times from thrombin generation analysis in the presence of lung homogenate) thrombin generation with addition of lung homogenate was reduced for both the analysis with and without addition of TF, PL, and Ca^2+^. These results suggested the presence of one or more inhibitors in lung and heart tissue with potential factors being glycosaminoglycans, components of the protein C pathway, or tissue factor pathway inhibitor (TFPI). While the latter cannot be ruled out with certainty, heparinase treatment of lung homogenates did not influence the inhibitory effect of lung tissue on thrombin generation (data not shown), suggesting no influence of endogenous glycosaminoglycans. Measuring thrombin generation in protein C-deficient human plasma, however, resulted in increased thrombin generation (e.g. higher ETP values compared to normal human pool plasma) after addition of lung homogenate (ETP: 1818 ± 176 nM.min), indicating that the observed reduction in thrombin generation after addition of lung homogenate to plasma was most likely dependent on a component of the protein C pathway (Figure 1C and 1F grey bars). The same was true for the addition of heart homogenate (ETP: 1619 ± 35 nM.min) (Figure 1F).

Since TM is one of the key factors in protein C activation we next analyzed organ homogenates from TM^Pro/Pro ^animals which carry a single amino acid functional mutation in the thrombin binding domain that markedly diminishes the protein C cofactor activity of TM [19]. Thrombin generation in normal pool plasma with addition of TM^pro/pro ^organs was comparable to levels obtained for human plasma alone (lung ETP: 1686 ± 209 nM.min, heart ETP: 1767 ± 200 nM.min, liver ETP: 2017 ± 66 nM.min), as well as wild-type organs added to protein C-deficient plasma (Figure 1D), indicating the presence of active TM in lung and heart homogenates. On the basis of these data it is likely that the inhibitory effect of specific organs in the thrombin generation assay is due to functionally active TM.

### Validation of the new method using a mouse endotoxemia model

To validate the new functional method for TF and TM activity in murine tissues a LPS-induced endotoxemia mouse model was used. One of the key characteristics of LPS-induced endotoxemia is the activation of coagukation as well as increased TF expression in lung tissue. Activation of coagulation upon LPS-induced endotoxemia was confirmed by measuring thrombin-antithrombin (TAT) complex levels 6 hours after lipopolysaccharide (LPS)-treatment of C57Bl/6 mice. LPS-stimulated mice showed a 20-fold increase in plasma TAT-levels (204.8 ± 123.3 ng/mL), compared to non-treated control animals (9.2 ± 7.7 ng/mL, p < 0.0001).

**Figure 3:**
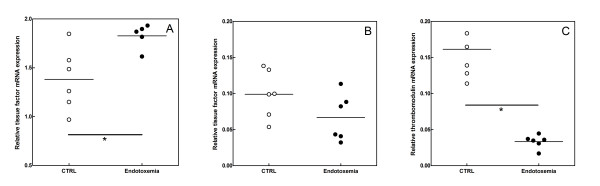
**TF (panels A and B) and TM (panel C) mRNA levels before (open circles) and 6 hours after LPS administration (filled circles) in lung (panel A and C) and heart (panel B).** mRNA levels are expressed as a ratio to GAPDH. Each dot represents the average of a triplicate measurement within one mouse. Horizontal lines indicate the mean expression level and * denotes p < 0.05.

To determine the procoagulant activity of LPS challenge at the organ level we compared TF activities in different organs from endotoxemic and control mice (Figure 2): TF activity in lungs of endotoxemic animals (25.8 ± 5.3 pM) was significantly higher than in lungs of control mice (12.7 ± 1.7 pM, p < 0.05), whereas the activity in other organs was comparable between both groups (Figure 2). In accordance, TF mRNA levels in lung tissue were significantly increased after endotoxemia (1.8 ± 0.1 vs. 1.4 ± 0.3, p < 0.02; Figure 3A), whereas the levels in other organs such as the heart (0.07 ± 0.03, Figure 3B) remained at a level comparable to controls (0.10 ± 0.03, p > 0.1) as expected.

**Figure 4:**
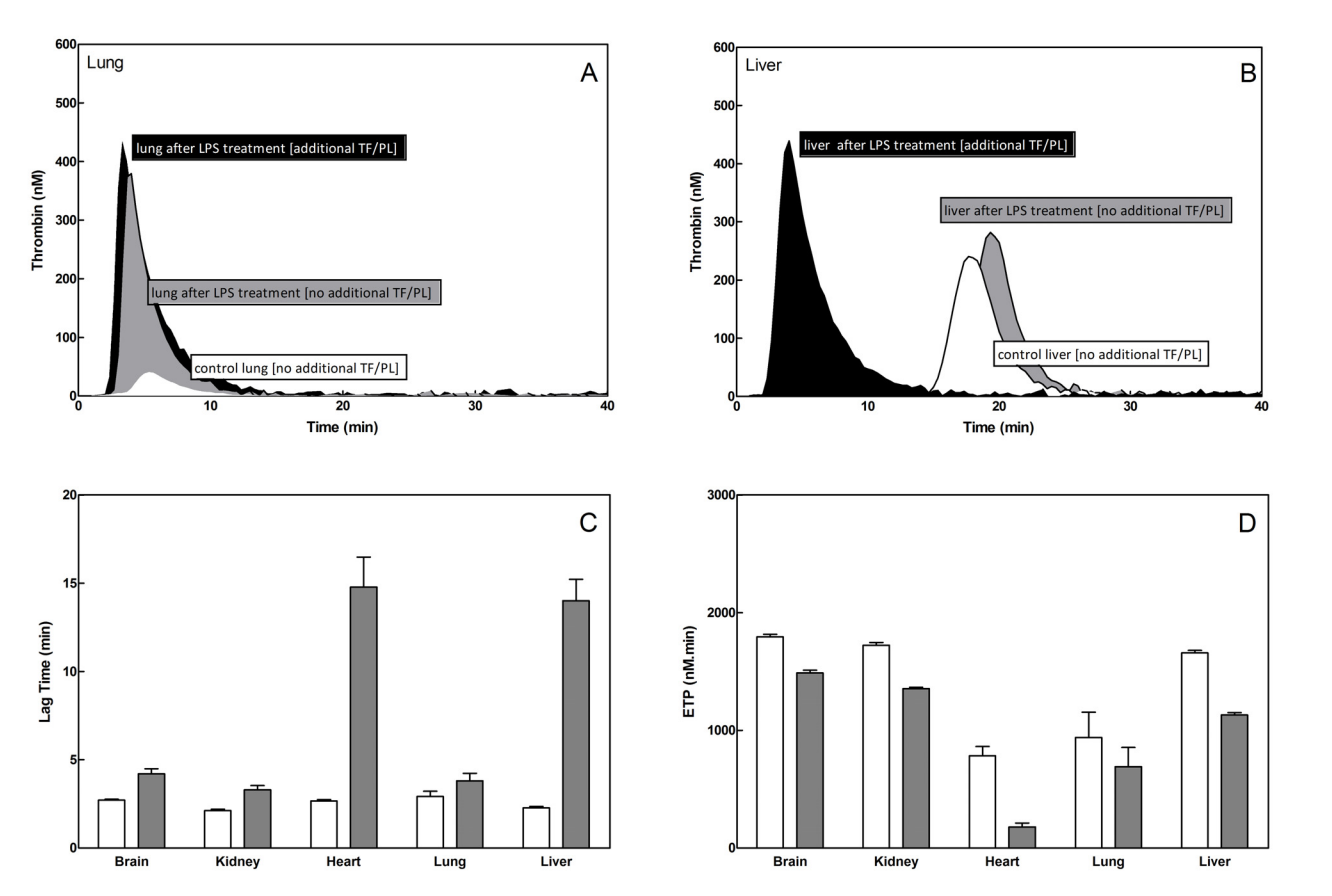
**Influence of LPS on thrombin generation triggered with additional TF (5 pM) and PL (4 μM) (open curves and bars) or without (grey curves and bars)**. Panel A and B: Typical thrombin generation curves recorded from normal human plasma in the presence of lung (A) or liver (B) homogenate 6 hours after LPS administration. White curve (○): control lung tissue without TF/PL. Grey curve (□): lung tissue after LPS-treatment without TF/PL. Black curve (△): lung tissue after LPS-treatment with TF/PL. Panel C and D: Lag times (C) and ETPs (D) derived from the thrombin generation curves recorded for normal human plasma in the presence of organ homogenates 6 hours after LPS administration. Bars represent mean ± SD from 6 tissue homogenates.

Comparing thrombin generation curves recorded in either the presence or the absence of TF, PL, and Ca^2+^, demonstrated a significant induction of thrombin generation for lung homogenates from mice subjected to LPS challenge (ETP: +TF: 940 ± 523, -TF: 692 ± 369 nM.min, Figure 4A), compared to controls (ETP: +TF:194 ± 159 nM.min, -TF: 103 ± 83 nM.min, p < 0.01, Figure 1F). These results are suggestive of attenuated inhibitory potential (e.g. decreased TM levels) or increased TF activity in lung tissue due to endotoxemia. The opposite influence of LPS was observed for heart tissue, which in the absence of PPP-reagent showed a significantly prolonged lag time after LPS challenge (15 ± 4 min, Figure 4C) compared to control heart tissue (11 ± 1 min, p < 0.05, Figure 4C). This later observation suggest decreased TF expression in the heart upon LPS induced inflammation, as observed previously by Luther et al. [28].

No changes were observed for brain (ETP: +TF 1795 ± 49 nM.min, -TF: 1488 ± 57 nM.min) and heart (ETP: +TF: 785 ± 90 nM.min, -TF: 179 ± 58 nM.min) (Figure 4D) from LPS-treated animals compared to control tissues (Brain ETP: +TF 1802 ± 43 nM.min, -TF: 1434 ± 60 nM.min; Heart ETP: +TF: 874 ± 74 nM.min, -TF: 144 ± 31 nM.min)(Figure 1F). Note that analyses were done both in the presence (+TF) and absence (-TF) of additional TF.

The increased thrombin generating activity of lung tissue after LPS treatment is suggestive for reduced availability of TM in the tissue homogenate, based on the aforementioned dependence of thrombin generation inhibition by TM in tissue; this conclusion is supported by the observed reduced TM mRNA expression in lung after LPS challenge (0.16 vs. 0.03, p < 0.01, Figure 3C). In contrast, we could not detect TM mRNA in other tissues.

### Effects of particulate matter on the procoagulant balance in lung tissue

**Figure 5:**
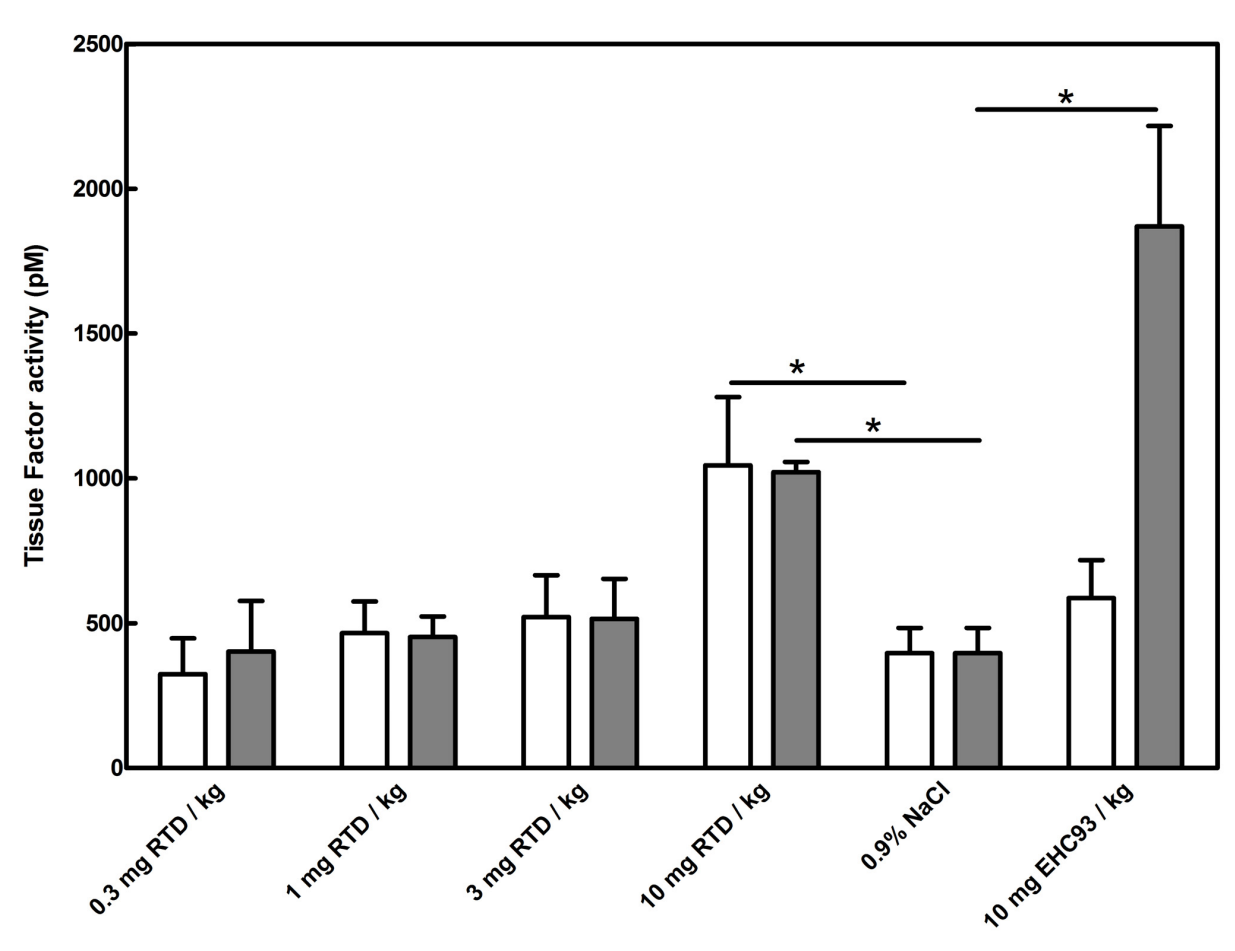
**TF activity in rat lung homogenates after 4 (white bars) and 48 hours (grey bars) exposure to PM.** Road tunnel dust (RTD) at final concentrations of 0.3, 1, 3, and 10 mg/kg BW was compared to saline, and EHC-93 at a final concentration of 10 mg/kg BW. Total protein concentration of the samples was 1 mg/ml. Bars indicate mean ± SD of n = 2 × 6 animals per group. * denotes p < 0.05.

Recent data showed enhanced expression of TF mRNA in lungs of spontaneously hypertensive rats after exposure to PM [29]. Here, we analyzed the pro- and anticoagulant activity in rat lung tissue at different time points (4 and 48 hours) after exposure to two types of PM: road tunnel dust (RTD) and urban dust (EHC-93). At 4 and 48 hours after exposure to RTD, TF activities in lungs showed a trend to increase with increasing concentrations of RTD. TF activity was significantly increased at the highest concentration of PM (10 mg RTD per kg BW) compared to saline instillation (at 4 hours: 1045 ± 472 pM vs. 397 ± 231 pM) (Figure 5). No differences in TF activity were observed between saline and EHC-93 (10 mg/kg) instillation at 4 hours after exposure (587 ± 260 pM), whereas TF activity was markedly increased (1870 ± 693 pM, p < 0.01) at 48 hours after exposure.

Thrombin generation (Table 1) was increased with rising concentrations of PM, with the difference between the dose of 10 mg/kg BW and saline control being significant at 4 hours (RTD: ETP 390 ± 148 nM.min, EHC-93: ETP 264 ± 64 nM.min vs. saline: ETP 186 ± 80 nM.min, p < 0.01) and 48 hours (RTD: ETP 1441 ± 289 nM.min, EHC-93: ETP 1719 ± 103 nM.min vs. saline: ETP 174 ± 61 nM.min, p < 0.0001). The increase in thrombin generation at 48 hours compared to 4 hours (with almost equal TF activities and lag times) strongly suggests a loss in TM activity at the 48 hrs time point.

**Table 1:** Influence of particulate matter exposed rat lung on the endogenous thrombin potential (ETP) in thrombin generation.

Treatment	ETP at 4 hrs (nM.min)	ETP at 48 hrs (nM.min)
0.3 mg RTD/kg	231 ± 105	103 ± 77
1 mg RTD/kg	264 ± 102	154 ± 128
3 mg RTD/kg	324 ± 102	288 ± 119
10 mg RTD/kg	390 ± 148*	1164 ± 199#
10 mg EHC-93/kg	264 ± 64*	1449 ± 32#
0.9% NaCl	175 ± 61	116 ± 60

RTD: Road tunnel dust environmental air pollution, EHC-93: urban dust environmental air pollution. * denotes significant difference compared to 0.9% NaCl treatment at 4 hrs. # denotes significant difference compared to 0.9% NaCl treatment at 48 hrs.

## Discussion

The interaction between coagulation and inflammation is a bidirectional process and the protein C pathway as well as TF plays key roles in this crosstalk [30,31]. In order to characterize the functional heterogeneity of procoagulant responses to inflammation in vivo, we undertook initial experiments in mice challenged with LPS, a commonly used inflammatory agonist. Endogenous as well as induced TF activities were relatively high in organs such as the brain and lungs and relatively low in organs like heart and liver, with a marked positive response in TF activity in the lungs 6 hrs after LPS challenge, which may be explained by increased production, as also indicated by the mRNA data. Expecting a procoagulant effect of organ-derived TF we tested the different mouse organs in a thrombin generation assay using human plasma as a source of prothrombin and other coagulation factors. We observed a marked heterogeneity in thrombin potentials, with the highest thrombin generation in brain, kidney and liver. An organ-dependent effect was observed, with as surprising observation the inhibitory effect of lung tissue on thrombin generation in plasma. Further experiments showed that this inhibitory effect of lung tissue was based on functional TM, as indicated by the fact that it was neutralized in protein C-deficient plasma as well as by replacement of wild type by TM^Pro/Pro ^lung tissue. Subsequent studies showed that the TM-associated activity was virtually lost after LPS infusion, demonstrating for the first time the functional loss in natural anticoagulant function *in vivo*.

Pro-inflammatory cytokines down regulate TM and EPCR in cultured endothelial cells [32] and our data show a suppression of TM mRNA in lung tissue. We were not able to detect significant TM mRNA levels in other tissues to document similar changes there, which may be related to density of endothelial cells and corresponding low amounts of mRNA. At the protein level there is clinical evidence for loss of circulating TM antigen levels and in the microvascular endothelium of children with meningococcal sepsis, which may be due to a combination of down regulation, release, and proteolysis [33,34]. Our data indicate that indeed, TM activity disappears from the pulmonary compartment although we cannot determine the reasons for this loss, other than diminished gene transcription. In the absence of antigen assays specific for mouse or rat TM we were not able to show concurrent changes in TM antigen levels.

Next we employed the coagulation activity assays to address the pro-inflammatory effects of PM in rats. This fraction of environmental pollutants has been associated with a number of pro-inflammatory effects in animal [23] and human cell culture [35] studies. In an unbiased search for mediators of PM induced inflammation in the lung Kooter and colleagues established among a panel of differentially expressed genes, an abundant expression of TF [29], which formed the basis for the present activity studies. Our data demonstrate that pulmonary instillation of PM induced TF activity as well as concurrent loss of TM associated activity in a concentration dependent manner. An increased TF and decreased TM activity in lungs, without being able to specify the cells involved, may contribute to a prothrombotic state, but more importantly increased amounts of thrombin that are less effectively quenched by TM, may facilitate increased interactions with other endothelial cell receptors including the family of G protein-coupled protease-activated receptors (PARs) [36]. Thrombin-mediated PAR signaling on endothelial cells results in a coordinated combination of responses and together with the reported anti-apoptotic [37] and pro-angiogenic [38] roles the cellular actions suggest that thrombin links haemostatic and inflammatory actions to vascular remodeling and angiogenesis.

The strength of our approach is that in contrast to previous studies we have addressed the functional changes, instead of the alteration in antigen levels, in coagulation activity inflicted by pro-inflammatory mediators in tissues from mouse and rat. Using the present procedures we were not able to distinguish which cell types express the observed pro- or anticoagulant activities. For instance, in the lung vascular cells including endothelial cells and smooth muscle cells may be largely responsible for the effects of TM, while to a lesser extent TF may be expressed by these cells. Epithelial cells may also contribute to TF expression. In the future the utilized assays may well be applied on tissue sections or isolated cells to address the issue of localization of pro- and anticoagulant activities.

In conclusion, the combination of assays for TF and thrombin generation revealed organ variability in procoagulant responses to specific inflammatory stimuli, indicated by alterations in TF and TM activities within the same organ. We showed that PM, a critical component of the industrialized environment, induced procoagulant activity in the lungs, which may ultimately contribute to pulmonary and cardiovascular disease.

## Abbreviations

CAT: Calibrated Automated Thrombogram; CTI: Corn trypsin inhibitor; EHC-93: urban dust PM sample collected from Environmental Health Centre in 1993, Ottawa, Canada; EPCR: Endothelial protein C receptor; ETP: endogenous thrombin potential; GAPDH: glyceraldehyde 3 phosphate dehydrogenase; HVCI: high volume cascade impactor; NPP: normal pool plasma; PL: phospholipids; PM: particulate matter; PPP: platelet-poor plasma; PUF: polyurethane foam; RTD: road tunnel dust; SHR: spontaneously hypertensive rats; TAT: Thrombin-antithrombin; TFPI: tissue factor pathway inhibitor; TF: Tissue Factor; TM: Thrombomodulin; WT: wild type.

## Competing interests

The authors declare that they have no competing interests.

## Authors' contributions

KM carried out the RNA analysis, drafted the manuscript, participated in the mouse studies and performed part of the statistical analysis. IK participated in the design of the study and performed rat exposure studies as well as part of the statistical analysis. RvO carried out the tissue preparations and thrombin generation analysis. DF participated in the thrombin generation analysis, tissue preparation and carried out the tissue factor analysis. KH participated in the design of the study and preparation of the manuscript. MG-N participated in the design of the study and performed the rat experiments. HtC conceived the study, and participated in its design and coordination. HS conceived the study, and participated in the mouse studies, statistical analysis and drafted the manuscript. All authors read and approved the final manuscript.
